# Alveolar cleft reconstruction using bone marrow aspirate concentrate and iliac cancellous bone: A 12-month randomized clinical study

**DOI:** 10.1007/s00784-023-05276-9

**Published:** 2023-10-05

**Authors:** Alshaimaa Ahmed Shabaan, Ahmad Salahuddin, Inass Aboulmagd, Reham Ragab, Khaled Amr Salah, Adel Rashid, Haytham Mohamed Ayad, Walaa Abd el Aty Ahmed, Shaimaa Mohsen Refahee

**Affiliations:** 1 Oral & Maxillofacial Surgery Department, Faculty of Dentistry, Fayoum University, Fayoum, 63511 Egypt; 2 Biochemistry Department, Faculty of Pharmacy, Damanhour University, Damanhour, Egypt; 3 Oral & Maxillofacial Radiology, Faculty of Dentistry, Fayoum University, Fayoum, 63511 Egypt; 4 Biomedical Informatics and Medical Statistics Department, Medical Research Institute, Alexandria University, Alexandria, Egypt; 5 Oral and Maxillofacial Surgery, Faculty of Dentistry, Cairo University, Cairo, 11111 Egypt; 6 Orthodontics, Faculty of Dentistry, Fayoum University, Fayoum, 63511 Egypt; 7 Oral and Maxillofacial Radiology, Faculty of Dentistry, Cairo University, Cairo, 11111 Egypt; 8 Biochemistry Department, Faculty of Pharmacy, Al-Ayen university, Nasiriyah, Iraq

**Keywords:** Bone grafting, Alveolar bone grafting, Bone marrow, Bone marrow cells, Growth factors

## Abstract

**Objective:**

This study aimed to compare the bone density and volume in patients with alveolar cleft reconstructions utilizing bone marrow aspirate concentrate with iliac graft versus iliac graft alone.

**Material and methods:**

Thirty-six patients with unilateral alveolar cleft were randomly allocated into either an intervention group receiving an iliac bone graft mixed with bone marrow concentrate or a control group receiving an iliac bone graft. Cone beam CT was obtained preoperative, 6 and 12 months postoperatively to assess the bone density of the graft and bone volume of the alveolar defect, and then, the bone loss ratio was calculated.

**Results:**

Bone volume and bone density demonstrated a statistically significant increase in the intervention group at 6 and 12 months. In contrast, the bone loss ratio decreased significantly in the intervention group throughout the follow-up period.

**Conclusion:**

A combination of bone marrow concentrate and iliac cancellous bone in alveolar cleft reconstruction may improve bone densities and volume in addition to decreasing graft loss rate.

**Clinical significance:**

Using of bone marrow aspirate concentrate will decrease the amount of the graft needed and decrease the ratio of bone loss at the grafted site by the time.

**Trial registration** ClinicalTrials.org (NCT04414423) 4/6/2020

## Introduction

Alveolar cleft reconstruction is needed to conserve the continuity of the arch, provide the maximum amount of bone support for the nose and permanent teeth eruption, and repair the residual oro-antral fistula [1, 2]. Alveolar cleft grafting is performed at various ages, but secondary grafting, performed between the ages of 9 and 12, is the most suitable and advantageous [3].

Due to its osteogenic, osteoinductive, and osteoconductive properties, the autogenous iliac bone graft is considered the “gold standard” for alveolar cleft repair [4–6]. However, the autogenous iliac bone graft is associated with a graft resorption rate of about 15–24% during the first six months [7–9]. In order to resolve those complications, different substances were combined with the iliac graft, such as platelet-rich plasma (PRP), fibrin glue, or bone morphogenic protein (BMP). Nevertheless, it was demonstrated that there was no difference between iliac grafts with or without these materials [10–12].

Various graft materials, such as allograft, xenograft, and synthetic bone grafts, have been utilized as an alternative to autogenous bone grafts. Allografts are expensive and associated with risks of bacterial contamination, viral transmission, and immunogenicity. Conversely, the main drawbacks of synthetic bone grafts are slow resorption and brittleness. A combination of hydroxyapatite and b-tricalcium phosphate, along with an autogenous bone graft, has been used to address these drawbacks. This combination can improve osteoconduction for bone formation and graft stability, leading to successful integration into a bone fusion mass [13, 14].

Bone marrow derivatives were considered alternative treatments of choice for alveolar cleft repair. Bone marrow aspirate concentrate (BMAC) is a concentration of bone marrow aspirate that contains a large number of hematopoietic stem cells (HSCs), mesenchymal stem cells (MSCs), endothelial progenitor cells, and growth factors as platelet-derived growth factor (PDGF), transforming growth factor- β (TGF-β), vascular endothelial growth factor (VEGF), epidermal growth factor (EGF), and basic fibroblast growth factor (bFGF) [15]. These cells and factors contribute to increasing bone volume, callus, and woven bone formation. Moreover, MSCs express VEGF, which induces neoangiogenesis and improves bone healing [15]. Although unconcentrated bone marrow aspirate contains all of these same elements, its effect on bone healing is dependent on both the number and concentration of these elements [16, 17].

Several studies used bone marrow concentrate in bone augmentation and fracture healing. According to Naujokat et al. [18], the addition of bone marrow concentrates to iliac bone graft enhances the bone quality and decreases the rate of bone resorption. On the contrary, Kühl et al. [19] illustrated that bone marrow concentrate had no effect on the dimension stability of deproteinized bovine bone during the first six months of follow-up.

No study has investigated the effect of the BMCS on autologous bone in the reconstruction of the alveolar cleft, despite its role in bone healing. Consequently, the present study targeted to assess the significance of BMAC mixed with iliac bone graft on the property and quantity of formed bone in unilateral alveolar cleft repair.

## Methods

### Study setting

 The included patients in this study were managed in the Oral and Maxillofacial Department of authors’ institutions between October 2020 and May 2023, for unilateral maxillary alveolar cleft grafting. The Ethics Committee at the Faculty of Dentistry, Cairo University approved the study (approval code: 27/9/20), and the methodology was posted on 4^th^ June 2020 on ClinicalTrials.org (ID: NCT04414423) https://clinicaltrials.gov/ct2/show/NCT04414423.

Before the study began, all patients’ guardians were given the opportunity to review the study’s objectives, harms, and significance before providing their informed consent. The study was completed in keeping with the Helsinki’s instruction [20] and the CONSORT 2010 [21].

### Study design and randomization (Fig. 1)

This is a prospective, randomized clinical trial with an allocation ratio of (1 : 1). The randomization sequence was an unstratified random block of sizes 2, 4, and 6 to confirm balance in the patients’ number allocated to each group. An investigator prepared a block randomization number list without clinical involvement in the trial.

**Fig. 1:**
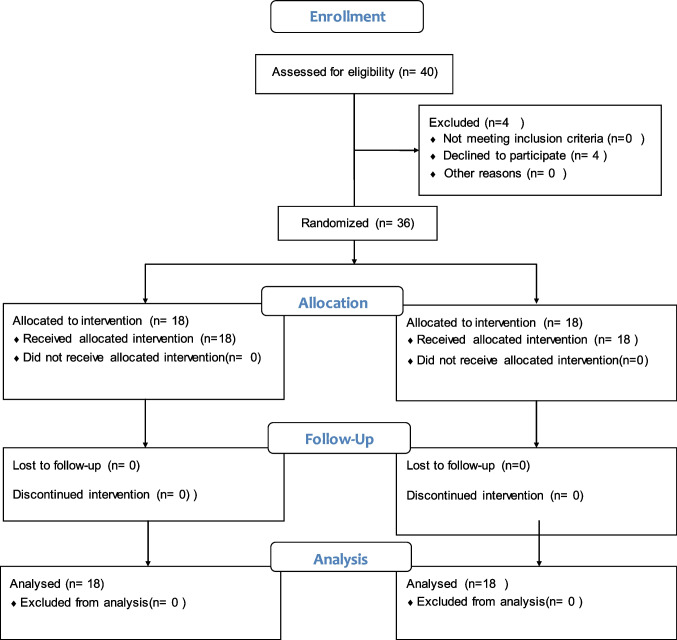
Consort follow chart

### Participants

The study included 36 patients with a unilateral maxillary alveolar cleft. The inclusion criteria were as follows: age ranged between 8 and 12 years, with good oral hygiene and general good health. Exclusion criteria were patients with syndromes associated with an alveolar cleft, local pathosis at the maxilla that may interfere with surgery, previous grafting attempts, or palatal fistulae.

The patients were randomly categorized to one of two parallel groups (1 : 1) based on the graft used as the intervention group (patients received iliac bone graft mixed with BMAC) and the control group (patients received iliac bone graft only). The outcome assessor and data analyzer were blinded to the allocation.

All patients were exposed to a carful clinical and orthodontic preoperative evaluation was performed. The radiographic assessment with cone-beam computed tomography (CBCT) (Planmeca Promax 3D classic, Planmeca, Finland) was carried out to measure the volume of the defect preoperatively.

### Operative procedures

All patients were operated on under general anesthesia. According to standard operating room procedures, intraoral and extraoral surgical sites were prepared.

### Preparation of BMAC (Fig. 2)

Before the graft surgery, the bone marrow aspirated from the posterior superior iliac crest by trochar that was inserted 2 cm laterocaudally. Ten millimeters of bone marrow was extracted into sterile plain tubes using a 20-ml heparinized syringe (1 ml of 5000 U/ ml heparin). The centrifugation was performed immediately consistent with the following protocol: 1^st^ centrifugation spin at 1200 *g* for 7 min, removal of the poor platelet plasma and extract of the cell concentrates in another plain tube for second centrifugation spin at 1200 *g* for 3 min. The pellet of the bone marrow cell concentrate was obtained suspended in 4 ml of plasma, which was then aspirated and mixed with harvested iliac bone graft in patients assigned to the intervention group [18].

**Fig. 2:**
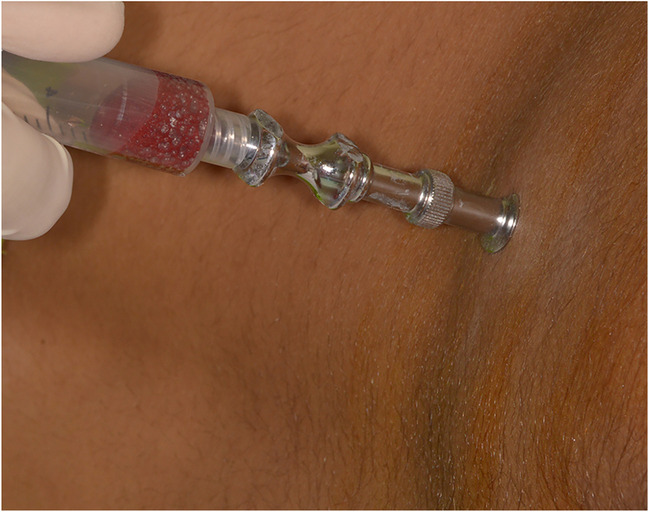
BMAC aspiration

### Surgical procedures (Fig. 3a–c)

The intraoral procedures and graft harvest were performed simultaneously by two groups of surgeons. On the alveolar cleft, an advancing full-thickness and four-corner flap was performed. The buccal and palatal flaps were reflected, and the scar within the cleft was excised. The flap was carefully dissected to ensure nasal lining watertight closure as well as free sliding for flap closure.

**Fig. 3:**
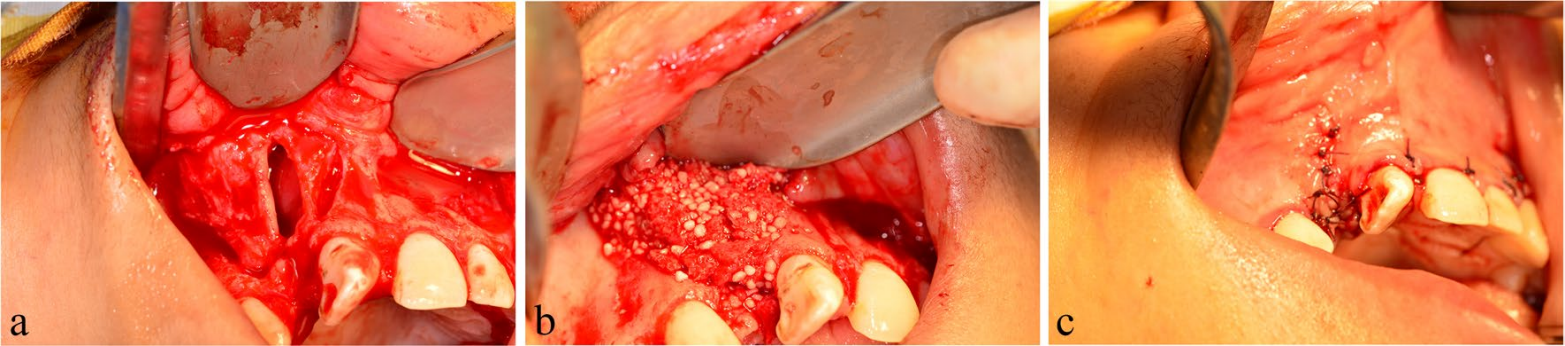
**a**–**c** The surgical procedures of alveolar cleft reconstruction with iliac graft and BMAC

A skin incision was performed 1 cm posterior to the anterior–superior iliac crest for iliac grafting. External oblique muscle and periosteum were reflected medially to uncover the anterior iliac crest. The cancellous graft was collected after trap door fenestration reflection. The wound was closed in three layers: muscle, subcutaneous, and skin [22] .

The cancellous bone was mixed with BMAC and packed for the intervention group to fill the alveolar defect. Conversely, cancellous bone was packed into the defect alone in the control group. The grafted area was covered with resorbable collagen membrane (Geistlich Bio-Gide®, Prof. Daniel Buser, Berne, Switzerland), and the labial flap was approximated and sutured over it by multiple interrupted sutures using a 4-0 Vicryl suture (Assut Medical Sarl, Pully-Lausanne, Switzerland). All patients and their guardians received the postoperative instruction as cold pack application for 20 min every 6 h, a soft diet was advised to be followed for the initial 48 h, and following the oral hygiene measures. Patients were instructed to take prophylactic antibiotic in the form of ampicillin sodium/sulbactam sodium 750 mg/12 h/5 days and ibuprofen 10 mg/kg every 8 h/ 3 days with further doses as needed. In addition, oxymetazoline hydrochloride 50% nasal drops (Afrin, MUP, Cairo, Egypt) 3 times/day/5 days was also included.

### Postoperative follow-up and outcome measurement

Postoperative clinical follow-up was performed once a week for the first month and once a month for the subsequent 12 months. The clinical follow-up was to determine complications, such as flap dehiscence, infection, hematoma, oronasal fistula, and nasal regurgitation.

Radiographic measurements were obtained preoperatively, 6, and 12 months postoperative to assess bone density, graft volume, and the rate of bone loss. The radiographic measurements were performed using standard CBCT scanning protocols with standard settings (90 kV, 6.3 mA, exposure period, 12 s, and voxel size, 0.2 mm). The same radiologist operating the CBCT equipment and Planmeca software performed the scanning (Romexis Planmeca, Planmeca, Finland). Radiographic evaluations were performed the day following surgery (as a baseline) as well as six and twelve months later.

**Fig. 4:**
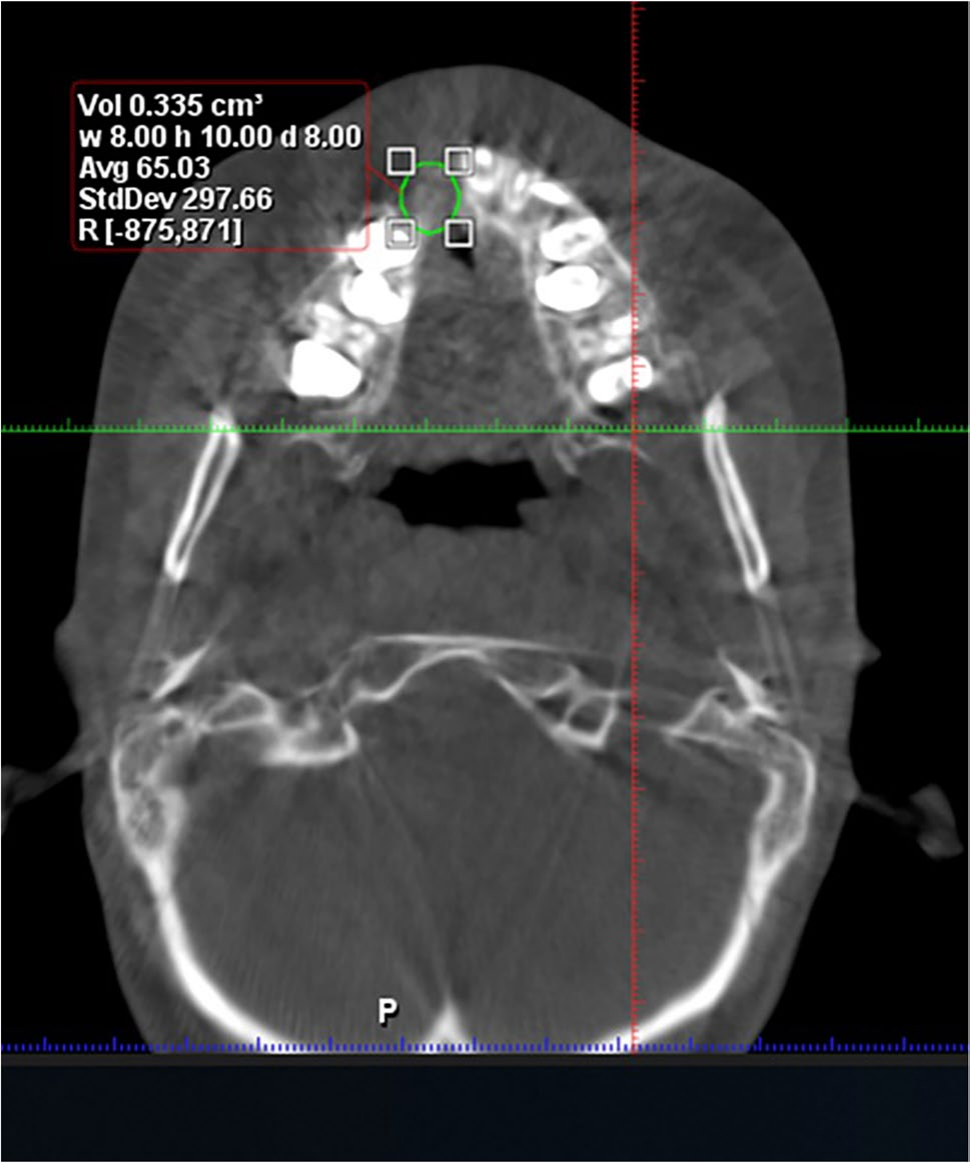
Bone volume and bone density measurement

The bone density (BD) was measured by Hounsfield unit (HU) values from the CBCT scans that were calculated by “Annotations Measure Rectangle.” Two blinded radiologists independently measured the averaged HU values to determine the final value (Fig. 4) [23] .

Regarding the bone volume (BV), it was measured by the freehand marking on the axial slices by the inherent feature of the software (Fig. 4) [23].

In addition, the bone loss percentage (BL%) was determined using the following equation:

1 $$(\mathrm{bone}\;\mathrm{volume}\;\mathrm{at}\;\mathrm{six}\;\mathrm{months}\;-\;\mathrm{bone}\;\mathrm{volume}\;\mathrm{at}\;12\;\mathrm{months})/\mathrm{bone}\;\mathrm{volume}\;\mathrm{at}\;\mathrm{six}\;\mathrm{months}\;\times\;100$$

### Sample size calculation

The primary outcome measure was bone density, as measured by HU. Using information from a prior study, a sample was calculated (STATA V16.0) with a study power of 80% at an alpha of 0.05 and effect size of 1.044 were used. The mean bone density of the intervention group was 360.82 HU and that of the control group was 384.03 HU. This revealed that a total of 32 patients would need to be taken into account. However, this study included a total of 36 patients (18 in each group) to compensate for any potential dropouts [24].

### Statistical analysis

Statistical analysis was performed using R version 4.1.1 associated with the following packages: (Tidyvers, rstatix, WRS2, and ggpubr). The normality distribution of our parameters was tested using the Shapiro test. Normally distributed data were presented using mean −/+ SD, while the skewed data were expressed as median (min–max). Categorical data were expressed as frequency and percentage. For binary variate analysis, either an independent *t*-test or the Mann–Whitney test was used according to the normality test. The two-way mixed ANOVA test was utilized to detect the interaction of group types on bone volume and density. The parameter difference is considered statistically significant if the *p*-value is less than 0.05. All confidence intervals were presented at the 95% level .

## Results

The study included 36 patients with unilateral alveolar cleft who were randomly categorized into two equal groups. In the intervention group, 18 patients received an iliac bone graft mixed with BMAC, while 18 patients received an iliac bone graft alone in the control group. On the right side, there were 20 clefts, and on the left, there were 16 clefts. The age of patients ranged between 8 and 11 years (Table 1).

**Table 1:** Demographic data in the two study groups

Variables		Intervention *n* = 18 (median (min–max))	Control *n* = 18 (median (min–max))
Age		9 (8–11)	9 (8–11)
Sex	Male 17 (47.22%)	9 (25%)	8 (22.22%)
	Female 19 (52.77%)	8 (22.22%)	11 (30.56%)
Hospital stay (days)		4	3.8
Site	Right (Rt) 15 (41.6%)	8 (44.4%)	7 (38.8%)
	Left (Lt) 21 (58%)	10 (55.5%)	11 (61.1%)

The postoperative wound healing was uneventful in both groups except for one case in the control group. The case showed dehiscence of the mesial releasing incision and was treated with vigorous mouthwash, and the patient was instructed to follow oral hygiene measures. The patients experienced moderate edema that subsided within 7 to 10 days postoperative, with no evidence of infection or oronasal fistula recurrence in all patients.

Patients required an average of 6 ± 3 days to walk normally and 25 ± 3 days to resume their normal life.

### Radiographic outcome

**Fig. 5:**
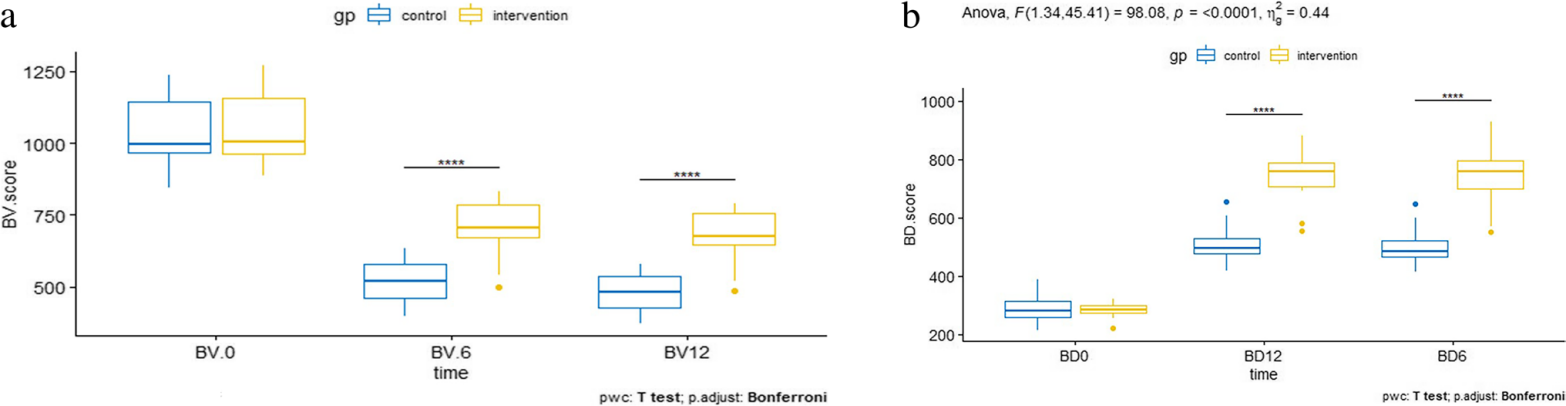
**a** The results of two-way mixed ANOVA of time vs. group on bone volume. **b** The results of two-way mixed ANOVA of time vs. group on bone density

As shown in Table 2, there is a statistically significant difference between the mean of BV and BD at 6 and 12 months postoperatively between the two groups (Fig. 5a, b). Using the Mann–Whitney test, the median BL% was statistically significantly decreased two times in the intervention group than in the control group (Table 2). The mixture of BMAC and iliac bone grafts utilized in the intervention group demonstrated superior outcomes in terms of bone volume, bone density, and bone loss ratio. In addition, the radiographic evaluation revealed that all alveolar defects in both groups were completely filled.

**Table 2:** Comparison of bone density, bone volume, and bone loss ratio in both groups

Variables	Intervention *n* = 18 (mean ± SD)	Control *n* = 18 (mean ± SD)	*p*
DV	1063 ± 123	1037 ± 112	0.509
BV6	709 ± 90	517 ± 72	<0.001*
BV12	681 ± 86	477 ± 65	< 0.001*
BD0	283 ± 23	289 ± 49	0.65
BD6	744 ± 93	502 ± 64	< 0.001*
BD12	741 ± 80	510 ± 64	< 0.001*
BL%	4 (3–5)	8 (6-9)	< 0.001*

*Significant (*p* < 0.05)

*DV* defect volume, *BV6* bone volume at 6 months, *BV12* bone volume at 12 months, *BD0* done density immediate postoperative, *BD6* bone density at 6months postoperative, *BD12* bone density at 12 months postoperative, *BL%* bone loss ratio

## Discussion

The surgical procedures of alveolar cleft reconstruction are controversial regarding the time, grafting materials, and surgical technique. Alveolar cleft grafting is performed at various ages, but secondary grafting, performed between the ages of 8 and 12 years (mixed dentition), is the most suitable and advantageous. The grafting in this age range gives a housing for canine eruption and allow the alignment of adjacent teeth [3]. This is in accordance with Oberoi et al. [25] who proved that the best time for grafting was between 9 and 11 years when the root of maxillary canine reach its ½ to 2/3 its length. This study is also in line with Elhaddaoui et al. [26] who reported the optimum time for alveolar cleft grafting was ranged between 8 and 12 years.

All patients involved in these study were unilaterally clefted with no significant difference regarding the cleft volum between the two groups to control the cofounders as the bone formation and the graft resorption ratio affected according to the cleft severity and cleft size [27].

Multiple graft material sources are used for cleft reconstruction, including autogenous bone grafts and allogenic and alloplastic grafts. There was a desire to develop an alternative graft material that could offer the benefits of bone graft with fewer complications and minimal graft loss rate in addition to improving the outcomes [28]. Bajestan et al. [29] used bone marrow stem cells with β-tricalcuim phosphate for alveolar ridge augmentation and found that autologous bone marrow stem cells can be used for bone regeneration of small and moderate defects. The current study targeted to assess the significance of BMAC mixed with iliac bone graft on the property and quantity of bone formation in unilateral maxillary alveolar cleft repair.

The BMAC is a source of MSCs, concentrated mononuclear cells (MNCs), platelet, cytokines, and growth factors which have anabolic and anti-inflammatory effects on the recipient tissue related to bone regeneration [30–33]. Moreover, BMAC had abundant interleukin-1 receptor antagonists that inhibit interleukin-1 catabolism and are responsible for other growth factors for symptomatic pain relief [34]. The preparation procedures for BMAC are based on bone marrow aspirate centrifugation to increase the cell concentration by 6–7-folds, improving the number of growth factors [35].

Pelegrine et al. [36] investigated the significance of the BMAC in preserving the alveolar ridges’ dimension stability after tooth extraction, and they concluded that the control and test groups had comparable amounts of mineralized bone. In contrast, Fontes Martins et al. [37] studied the mineralized tissue level and bone markers’ expression in sockets grafted with platelet-rich fibrin and BMAC. They found that extraction sockets that received the combination of bone marrow aspirate concentrate and PRF showed more mature bone and higher expression of osteoclastin. This result may be attributed to the process of centrifugation used in BMAC, resulting in a graft with more osteogenic characteristics that maximized the bone formation process.

The present study showed no dehiscence, infection, or oronasal fistula recurrence in all patients in the BMAC group. This finding aligns with Elhadidi et al. [38], who illustrated that alveolar cleft grafting using bone marrow stem cells concentrate/platelet-rich fibrin shown to have better soft tissue healing, lower dehiscence rates, and lower pain edema ratings at the operative site.

In this study, an autogenous particulate iliac graft was used as it has osteogenic, osteoinductive, and osteoconductive properties [4, 5]. In addition, it is more easily incorporated into the grafted site, and the graft spicules can exfoliate without impacting the rest of the graft. Furthermore, the particulate bone graft demonstrated favorable results regarding the dehiscence of the wound [39]. In contrast, the use of corticocancellous bone graft showed a higher incidence of wound dehiscence (16.6%) that could jeopardize the alveolar cleft graft outcome [40]. Despite all the advantages of particulate iliac bone graft, it still had a high resorption rate that decreased its quality for bone grafting, which may be attributed to its trabecular configuration [39, 41]. Therefore, in the present study, the particulate bone graft was mixed with BMAC to improve its outcomes.

Regarding the bone volume, there was a significant difference between the mean of BV of the intervention and placebo groups after six months (*p* < 0.001). Moreover, there was a significant difference between the mean of BV of the two groups after 12 months ( *p* < 0.001). These results are inconsistent with Elhadidi et al. [38], who used the BMAC during the consolidation period of distraction osteogenesis and concluded that there was a nonsignificant increase in bone volume between the intervention and control groups. On the contrary, Peleigou et al. [42] evaluated the management of 60 patients with atrophic non-union tibia with BMAC. They concluded that the volume of the mineralized callus had a positive correlation with the number and concentration of fibroblast colony-forming units in the graft. In addition, Fengzhou et al. [43], who used bone marrow stem cells with β-tricalcuim phosphate in the reconstruction of alveolar cleft, approved that the bone formation was significantly reduced at six months and no significant changes occur at 12 months.

Concerning bone density, the present study reported a statistically significant difference in the BD parameters between the two groups after six months and 12 months (*p* < 0.001). This finding is in agreement with Fontes et al. [37], who studied the mineralized tissue level and bone markers’ expression in sockets grafted with platelet-rich fibrin and BMAC. They found that the extraction socket of the BMAC and PRF groups showed more mature bone and higher expression of osteoclastin. Contrarily, Elhadidi et al. [38] concluded that bone density showed a nonsignificant increase in the study group compared to the control group. Similarly, Mossaad et al. [44] determined the quality of regenerated bone at the unilateral alveolar cleft region after using bone marrow and platelet-rich membrane grafting technique using dual-energy X-ray bone density scan (DEXA). They concluded that there was no statistically significant difference in DEXA bone mineral content measurements between the cleft and standard sides.

Relating to the bone loss ratio rate, there was a significant difference between the intervention and control groups (*p* < 0.001). These results align with Naujokat et al. [18], who proved the efficacy of BMAC in decreasing the rate of bone loss in alveolar ridge augmentation.

Regarding the previous study, cleft severity, defect size, and patient age would affect the study outcomes. Accordingly, there were attempts to control the different cofounders in the current study as the eligible patients’ age were 8–12 years with unilateral alveolar cleft and no difference regarding the volum between the two groups and this was considered as a point of study strength.

The current study had a number of limitations. Only unilateral alveolar defects were involved in the analysis. As the local conditions and resorption rate differed between bilateral and unilateral clefts, the reconstruction of the bone may be different. In addition, the present study did not include patients with variable defect volumes, which would have allowed subgroup analysis and shed light on the graft behavior in different defect volumes.

## Conclusion

Based on the results of the present study, we can conclude that a combination of BMAC and autogenous iliac bone graft in alveolar cleft grafting may improve bone densities and volume. The combination of BMAC with other graft materials requires additional clinical investigation. In addition, further studies comparing between bone morphogenic protein and BMAC with iliac graft are recommended.

## Data Availability

For data protection, the datasets of the current study can be gotten from the corresponding author.

## Abbreviations

*BD*: bone density
*bFGF*: basic fibroblast growth factor
*BL%*: bone loss ratio
*BMAC*: bone marrow aspirate concentrate
*BMP*: bone morphogenic protien
*BV*: bone volume
*CBCT*: cone-beam computed tomography
*DEXA*: dual energy x-ray bone density scan
*EGF*: epidermal growth factor
*HSCs*: hematopoietic stem cells
*HU*: Hounsfield unit
*IGF-1*: insulin-like growth factor 1
*MNCs*: mononuclear cells
*MSCs*: mesenchymal stem cells
*PDGF*: platelet-derived growth factor
*PRP*: platelet-rich plasma
*TGF-β*: transforming growth factor-β
*VEGF*: vascular endothelial growth factor
